# Identifying Factors for Optimal Development of Health-Related Websites: A Delphi Study Among Experts and Potential Future Users

**DOI:** 10.2196/jmir.1863

**Published:** 2012-02-14

**Authors:** Francine Schneider, Liesbeth van Osch, Hein de Vries

**Affiliations:** ^1^CAPHRIDepartment of Health PromotionMaastricht UniversityMaastrichtNetherlands

**Keywords:** Health promotion, Internet interventions, health behavior change, optimal development, Delphi study, experts, users

## Abstract

**Background:**

The Internet has become a popular medium for offering tailored and targeted health promotion programs to the general public. However, suboptimal levels of program use in the target population limit the public health impact of these programs. Optimizing program development is considered as one of the main processes to increase usage rates.

**Objective:**

To distinguish factors potentially related to optimal development of health-related websites by involving both experts and potential users. By considering and incorporating the opinions of experts and potential users in the development process, involvement in the program is expected to increase, consequently resulting in increased appreciation, lower levels of attrition, and higher levels of sustained use.

**Methods:**

We conducted a systematic three-round Delphi study through the Internet. Both national and international experts (from the fields of health promotion, health psychology, e-communication, and technical Web design) and potential users were invited via email to participate. During this study an extensive list of factors potentially related to optimal development of health-related websites was identified, by focusing on factors related to layout, general and risk information provision, questionnaire use, additional services, and ease of use. Furthermore, we assessed the extent to which experts and potential users agreed on the importance of these factors. Differences as well as similarities among experts and potentials users were deduced.

**Results:**

In total, 20 of 62 contacted experts participated in the first round (32% response rate); 60 of 200 contacted experts (30% response rate) and 210 potential users (95% response rate) completed the second-round questionnaire, and 32 of 60 contacted experts completed the third round (53% response rate). Results revealed important factors consented upon by experts and potential users (eg, ease of use, clear structure, and detailed health information provision), as well as differences regarding important factors consented upon by experts (eg, visual aids, self-monitoring tool, and iterative health feedback) or by potential users only (eg, bread crumb navigation and prevention of receiving spam).

**Conclusions:**

This study is an important first step in determining the agreed-upon factors that should be taken into account when developing online health promotion programs. The public health impact of these programs will be improved by optimizing the development process in line with these factors.

## Introduction

Worldwide, more and more people are accessing the Internet in search of health-related information [1]. It is estimated that globally a minimum of nearly seven million health-related Internet searches are conducted daily [2]. Since Internet penetration rates are still expanding, with currently almost two billion people having access to the Internet, the number of health-related searches is also expected to increase [3]. Therefore, the Internet is a promising channel for offering a broad range of health-related information, such as background information on health, treatment information, medication information, and health behavior information [1].

 Due to this high level of accessibility and its potential to reach large numbers of people [3], the Internet has also become a popular medium in the field of health promotion for offering tailored and targeted health promotion programs [4,5]. As a consequence, in recent years positive effects of online interventions applying computer-tailored techniques have been reported addressing different health behaviors [6], such as physical activity [7,8], nutrition [9,10], smoking cessation [11-14], and alcohol consumption [15,16]. Although these tailored interventions are very promising and have proven to be effective, actual reach is failing to live up to the high expectations [17-22]. Since the public health impact of interventions is determined not only by their efficacy but also by their levels of exposure in the target group [23,24], it is imperative to put effort into optimizing the level of exposure to Internet-delivered lifestyle interventions.

Successful exposure is partly defined by the level of first-time access of the intervention, also referred to as *first use* or adoption ([19,24,25]. Besides first use, *prolonged use* of the intervention is essential. That is, engaging users in the intervention for a substantial amount of time fosters their knowledge of its content and involvement in its effective components, which consequently increases the chances of health behavior change [17,26]. Since health behavior change is a complex and continuous process, achieving sustained behavior change depends on both the intensity of the intervention and the number of times the intervention is visited [27,28]. Due to this high dose–response relationship, ensuring adherence or *sustained use* of the program is essential to further maximize its effect on subsequent health behavior change [29,30]. Both prolonged and sustained use of the intervention can be influenced not only by user characteristics (eg, demographic characteristics and motivation to use the intervention [17,31,32]) but also by specific strategies to increase adherence (eg, sending periodic reminders [33,34]) and by intervention characteristics (eg, appearance and content of the intervention [33,35]).

As online behavior change interventions are delivered to the public by using a website or Web-based program, development of the website or program refers to composition of the actual intervention, and requires careful composition of the complete website or program it is embedded in. The website as a mode of delivery is described in the Internet intervention model as consisting of 8 main areas [35]: appearance (eg, the organization of information), behavioral prescriptions (eg, instructions on how to achieve behavior change), burdens (eg, poor navigation applications), content (eg, treatment information), delivery (eg, use of animations, audio, or graphics), message (eg, credibility and likability of the source), participation (eg, degree of interaction or the use of rewards), and assessment (eg, measuring needs of users or adjusting content to personal wishes). Adjusting these characteristics enables tailoring of the website to special needs of the population under consideration. All individual characteristics in these areas should therefore be kept in mind while developing the program.

The primary characteristics of an intervention are determined during its development process. According to diffusion theory and social marketing principles [25,36], program development is one of the main processes to influence adoption rates of a new product (eg, a new website or online intervention). Besides including experts in the development process, it should also be done in accordance with the needs and wishes of the target group [36]. By considering and incorporating the opinions of potential future users, involvement is expected to increase, consequently resulting in increased appreciation of the intervention, lower levels of attrition, and higher levels of prolonged and sustained use [37]. A new intervention should, therefore, be developed in close collaboration with the target group.

 Although many studies have investigated the effectiveness and appreciation of numerous components of websites that deliver health behavior interventions, results have been inconclusive. Some studies recommend using a multimedia approach [38] or the use of interactive tools, such as surveys, quizzes, and games, whereas other studies seem to contradict these findings [5]. Systematically studying different elements of websites is a very extensive and time-consuming process. Even though some elements of websites should be incorporated at all times, such as accurate and comprehensible information [39], a lot of effort should still be put into making other elements operational [35].

 To date, studies on development of health-related websites have not included perspectives of both experts and users. Hence, this study was a first attempt to identify which elements on health-related websites are perceived as necessary and preferable by these two groups. The current study thus included both experts in the fields of health promotion, health psychology, e-communication, and technical Web design and potential future users. By including elements that are perceived as important by both groups while developing the website, developers may optimize exposure rates of the program [36]. Furthermore, involving different groups of experts will lead to more diverse information regarding health communication and behavior change as well as technical information regarding website development. To identify the potential factors that are related to optimal development of health-related websites, we conducted a three-round Delphi study. The specific aim of the study was to identify factors that are associated with optimal development of health-related websites. Besides identifying these factors, we investigated the degree to which experts and potential future users agreed on the importance of the factors.

## Methods

We conducted a three-round Delphi study through the Internet. Due to its systematic nature, a Delphi study is considered to be an appropriate method to derive consensus on health-related issues for which scientific evidence is incomplete or scarce by involving a representative panel of experts [40]. Also, because after each round feedback on group results is provided, the iterative approach allows participants to adjust their opinions when needed. Finally, the structure of the study guarantees anonymity of the participants, thereby preventing conformity biases [41,42].

The current Delphi study consisted of two substudies. The first substudy (study 1a), including only experts, consisted of three rounds. The first round aimed at providing a list of potential factors related to optimal development of health-related websites by means of an open-ended questionnaire. Next, for the second round, experts were invited to rate the importance of all factors identified in the first round by using a structured questionnaire. Finally, a third round enabled experts to reevaluate their opinions by providing controlled feedback regarding group mean scores, thereby producing consensus. The second substudy (study 1b) included only potential future users and consisted of one round. This round resembled the second round of the first substudy and allowed users to rate the importance of those factors that were identified by experts in the first round by using the same structured questionnaire. We compared second-round results from both studies in order to identify potential differences between experts and potential users regarding the importance they placed on factors related to optimal design.

### Study 1a: Experts

#### First Round

##### Procedure and Participants

For the first round, we selected experts from the fields of health promotion, health psychology, e-communication, and technical Web design to obtain a variety of insights from researchers with both theory-based and more practice-based backgrounds. Invited experts with a theory-based background were all first or second authors on scientific papers in the field of eHealth and eHealth promotion published between 2000 and 2009. We used database searches in PsychINFO and Medline to identify experts and to examine reference lists from related papers, book chapters, review studies, and conference abstracts. We selected experts with a practice-based background on the basis of their publications, but also by approaching our own network and by asking responding experts to provide names of important experts in the field.

This resulted in a list of 62 experts who were invited by email to participate in all three rounds of the Delphi study. The email contained detailed information on the goal and study procedure, as well as a link referring them directly to the first-round questionnaire. Nonresponders received a reminder email after the 3-week response period expired. A total of 20 experts (32% response rate) responded to the invitation.

##### Questionnaire

The first-round questionnaire consisted of 7 open-ended questions. The questions pertained to different subjects related to the development of health-related websites: (1) optimal layout, (2) type of general information provided, (3) type of health risk information provided, (4) ease of use, (5) use of visual aids, and (6) additional information provided. Health-related websites often provide questionnaires to allow visitors the opportunity to assess their own current health status. To provide an accurate update of their health status these questionnaires often tend to be extensive and therefore sensitive to early dropout. Therefore, we also included one open-ended question to gain more insight into factors that contribute to completion of the questionnaires often provided on health-related websites (7). Health-related websites were defined as websites aiming at assisting people to adopt a healthier lifestyle, by offering them important and diverse information regarding health and health-related behaviors (eg, physical activity, smoking, alcohol consumption, and nutrition).

##### Data Analysis

Responses of experts were analyzed, resulting in an extensive list of potential factors related to optimal development of health-related websites. Two researchers independently listed all unique factors and combined similar responses into 1 factor. For those factors on which no agreement was obtained, a third researcher was approached to give a decisive answer.

#### Second Round

##### Procedure and Participants

Experts participating in the first round were also invited to participate in the second round. We selected an additional 180 experts by means of the same strategies used for the first round.

A total of 200 experts received an email inviting them to participate in the second and third rounds. The invitation contained a link that directed experts to the second-round questionnaire. Nonresponders received two reminder emails: after 3 and after 5 weeks. A total of 60 experts (30% response rate) responded to the invitation (Table 1).

**Table 1:** First-, second-, and third-round rates of responses of experts

Field	First round (n = 62)		Second round (n = 200)		Third round (n = 60)	
	Invitations	Response (%)	Invitations	Response (%)	Invitations	Response (%)
Health promotion	16	7 (44%)	60	18 (30%)	18	10 (56%)
Health psychology	16	5 (31%)	60	14 (23%)	14	8 (57%)
E-communication	15	5 (33%)	40	12 (30%)	12	5 (42%)
Technical Web design	15	2 (13%)	40	16 (40%)	16	9 (56%)
Total	62	20 (32%)	200	60 (30%)	60	32 (53%)

##### Questionnaire

The second-round questionnaire was composed based on the factors identified by experts in the first round. This resulted in a questionnaire consisting of 85 structured questions. Experts were asked to rate these factors on a 7-point Likert scale ranging from 1 (not at all important) to 7 (extremely important).

##### Data Analysis

The closed-ended questions were analyzed following the standards for analyzing data for a Delphi study, by calculating median scores, also referred to as the 50^th^ percentile score, to determine the importance of the various factors. Furthermore, the interquartile range (IQR) was calculated to assess the degree of agreement between the experts on the importance of the factors [31,41,43]. The IQR represents the distance between the 25^th^ and the 75^th^ percentiles, with a small value indicating a higher degree of agreement. An IQR smaller than 1 is considered to indicate good consensus on a 7-point scale as used in the present study and means that more than 50% of all cases fall within 1 point of one another [44]. To deduce those factors that were considered to be either very or extremely important by the majority of experts (IQR < 1), the cut-off point for importance was a median score of ≥6.

#### Third Round

##### Procedures and Participants

All experts participating in the second round (n = 60) were invited to participate in the third and final Delphi round, using the same procedure as in the previous round. A total of 32 experts (53% response rate of second-round participants) responded to the invitation (Table 1).

##### Questionnaire

The third-round questionnaire was an adapted version of the second-round questionnaire, containing only those factors (n = 27) on which no consensus was obtained during the second round (IQR > 1). In line with the Delphi method, additional feedback on second-round group results (median and IQR) was provided, enabling experts to rerate their answers.

##### Data Analysis

The degree of agreement and consensus among experts was measured by computing median scores and IQRs.

### Study 1b: Potential Future Users

#### Round 2

##### Procedures and Participants

Potential future users were recruited through an independent, commercial Dutch Internet research panel [45]. From this panel, consisting of approximately 20,000 members, a sample of 220 members were invited to participate in this study. Potential users were invited to take part only in the second round of this Delphi study, since the main goal was to compare users’ opinions with experts’ opinions and not to force consensus among users on the different factors. Respondents from this panel were rewarded for their participation in this study in accordance with the standards of the consumer panel (approximately €5). All participants received an email invitation informing them about the goal and content of the study. The invitation also contained a link that directed participants to the questionnaire. A total of 210 potential future users (95.5% response rate) responded to the invitation (Table 2).

**Table 2:** Demographic profile of potential future users (n = 210)

Demographic characteristic		%	n
**Age (years)**			
	Range	19–65	
	Mean (SD)	46.49 (47.00)	
**Gender**			
	Male	51	106
	Female	49	104
**Ethnicity**			
	Native	98	206
	Nonnative	2	4
**Education level**			
	Low	11	24
	Medium	59	123
	high	30	63
**Income**			
	Less than average	29	61
	Average	26	54
	More than average	45	94
**Internet use: work or study**^a^			
	Frequent	55	116
	Average	12	26
	Infrequent	10	20
	Not applicable	23	48
**Internet use: private use**^a^			
	Frequent	96	202
	Average	3	6
	Infrequent	1	2
**Internet use: health purposes**			
	Frequent	21	45
	Average	69	144
	Infrequent	10	21

^a^ Frequent user: >3 times/week; average user: every week, but not >3 days/week; infrequent user: <1/week.

##### Questionnaire

The questionnaire assessed demographics such as gender, age, ethnicity, education level, and income. In addition, participants were asked to indicate how often they used the Internet for work or study purposes (1, [almost] never; 6, every day), for private use (1, [almost] never; 6, every day), and for finding information on health-related topics (1, never; 6, very often). For the questions assessing Internet use for work and private purposes, new categories were composed: frequent user (>3 times a week), average user (every week, but not >3 days a week), and infrequent user (<1 a week).

Furthermore, the questionnaire contained all factors identified by experts in the first round of study 1a, resulting in a questionnaire consisting of 92 questions. Potential future users were asked to rate the importance of all factors on a 7-point Likert scale ranging from 1 (not at all important) to 7 (extremely important).

##### Data Analysis

Data were analyzed according to the same principles used for the second round of study 1a, by calculating median scores and IQRs. Differences in consensus between the expert group and potential future users were analyzed using Wilcoxon signed rank sum tests. Similarities and differences in second-round results between experts and potential future users were further analyzed by using multivariate analysis of variance.

## Results

### Study 1a: Experts

We used experts’ responses from the first round to compose the questionnaire for the second round, and results of the first round are therefore shown as question items. We grouped items with regard to layout, general information content, health risk information content, ease of use, questionnaire completion, visual aids, and additional services. This resulted in a list of 85 factors thought to be related to optimal development of health-related websites. There were 10 factors in the layout category, 11 in general information content, and 16 in health risk information content. A total of 18 factors were mentioned as facilitating ease of use. The remaining factors referred to questionnaire completion (11), visual aids (6), and additional services (13). An overview of all results is given in Multimedia Appendix 1.

#### Consensus

During the second round, consensus was reached (IQR ≤ 1) on 57 factors. After experts rerated their answers during the third round, aided by feedback on second-round group results, consensus was obtained on another 6 factors. In total consensus was obtained on 74% of all factors.

#### Importance

Experts identified 33 factors as being very or extremely important (median ≥ 6): (1) layout, with 5 factors (50% of layout-related factors, eg, professional appearance and use of color), (2) general information provision, with 4 factors (36% of general information-related factors, eg, information on pros and cons of a healthy lifestyle, and tailored information on health), (3) health risk information provision, with 3 factors (19% of health risk-related factors, eg, information on skills that help to decrease health risks and personal advice on how to decrease health risks), (4) ease of use, with 10 factors (56% of factors related to ease of use, eg, availability of an easy log-in procedure and a clear navigation structure), (5) questionnaire completion, with 6 factors (55% of factors related to completion, eg, provision of a progress bar and provision of an option for partial completion), and (6) additional services, with 5 factors (38% of factors related to additional services, eg, provision of a self-monitoring tool and iterative feedback).

 Although consensus was obtained on 3 factors relating to the provision of visual aids (eg, provision of cartoons, pictures, and graphical representations of relevant information), these factors had respective median scores of 4, 5, and ,5 and thus were not considered as extremely or very important. Combining the results on importance and obtained consensus, we can conclude that consensus (IQR ≤ 1) was obtained on the importance (median ≥ 6) of 24 factors (Table 3).

**Table 3:** Median scores for important factors in health-related Internet sites on which consensus was obtained by experts, potential users, or botha

Factor		Second round				Third round	
		Experts (n = 60)		Users (n = 120)		Experts (n = 32)	
		Median	IQR^b^	Median	IQR	Median	IQR
**Which factors determine optimal layout?**							
	1. User friendly^c^	7	1	6	2	NA^d^	NA
	2. Lively appearance^c^	6	1	5	1	NA	NA
	3. Use of visual materials, such as pictures, videos, and graphics^c^	6	1	5	1	NA	NA
	4. Use of color^c^	6	1	5	2	NA	NA
	5. Professional appearance^e^	6	2	6	1	6	1
**What kind of general information should definitely be provided on health?**							
	6. Information on how to attain a healthy lifestyle^c^	6	1	6	1	NA	NA
	7. Information on pros and cons of a healthy lifestyle^c^	6	1	5	1	NA	NA
	8. Information on health risk behaviors^c^	6	1	5	1	NA	NA
	9. Personal tailored information on health^c^	6	2	5	1	6	1
**What kind of health risk information should definitely be provided?**							
	10. Information in the form of visual aids, eg, graphs^c^	6	1	5	1	NA	NA
**Which factors determine optimal ease of use?**							
	11. Clear structure^f^	7	1	6	1	NA	NA
	12. Availability of an easy log-in procedure^f^	7	1	6	1	NA	NA
	13. Use of comprehensive language^c^	7	1	6	2	NA	NA
	14. Clear navigation structure^c^	7	1	2	2	NA	NA
	15. Simple site design^f^	6	1	6	1	NA	NA
	16. Availability of a helpdesk^c^	6	1	5	2	NA	NA
	17. Availability of a function to customize the site for personal needs^c^	6	1	4	2	NA	NA
	18. Availability of bread crumb navigation^e^	5	2	6	1	5	2
	19. Availability of contact information for developers^c^	6	2	5	1	6	1

**Which factors determine whether visitors complete questionnaires provided on the sites?**							
	20. Clearly structured questionnaire with clear headings and subheadings^f^	6	1	6	1	NA	NA
	21. Progress bar^f^	6	1	6	1	NA	NA
	22. Opportunity to stop completion and proceed at a later time^f^	6	1	6	1	NA	NA
	23. Information on personal benefits of completion^c^	6	1	5	1	NA	NA
	24. Information on relevance of questionnaire completion^c^	6	1	5	1	NA	NA
	25. Guarantee that completion will not result in receiving spam^e^	6	2	7	1	5	1
	26. Use of original questions^e^	4	1	6	1	NA	NA
**What additional services should be provided?**							
	27. Search engine^e^	5	1	6	1	NA	NA
	28. Opportunity to print or download relevant information^c^	6	1	5	1	NA	NA
	29. Opportunity for regularly revisiting the site^c^	6	1	5	1	NA	NA
	30. Tool to self-monitor personal health behavior change^c^	6	1	5	2	NA	NA
	31. Iterative feedback during revisits to assess users against their own previous performances^c^	6	1	5	2	NA	NA
	32. Privacy statement^c^	6	2	5	2	6	1

^a^ Only experts were included in the third round. Statements on which consensus was obtained in the second round were excluded from the third round and results are therefore missing. Only factors on which consensus was obtained, either by experts or users, or both, are displayed (interquartile range ≤ 1).

^b^ Interquartile range.

^c^ Factors on which consensus was obtained only by experts.

^d^ Not applicable.

^e^ Factors on which consensus was obtained only by potential users.

^f^ Factors on which consensus was obtained by both experts and potential users.

### Study 1b: Potential Future Users

#### Consensus

Potential future users reached consensus (IQR ≤ 1) on 60 of the 85 factors (71%). An overview of all results is given in Multimedia Appendix 1.

#### Importance

A total of 17 factors were identified as being very or extremely important (median ≥ 6). These factors were mapped into different categories: (1) layout, with 3 factors (30% of factors related to layout, eg, limited amount of distractions and user friendliness), (2) general information provision, with 1 factor (9% of factors related to general information; information on how to attain a healthy lifestyle), (3) ease of use, with 7 factors (39% of factors related to ease of use, eg, availability of an easy log-in procedure and use of comprehensive language), (4) questionnaire completion, with 3 factors (55% of factors related to questionnaire completion, eg, provision of a progress bar and provision of an option for partial completion), and (5) additional services, with 5 factors (38% of factors related to additional services, eg, provision of a self-monitoring tool and iterative feedback).

 Although consensus was obtained on 14 factors relating to ease of use (eg, information on skills that help to decrease health risks, and personal advice on how to decrease health risks), all these factors had median scores of 5 and were not considered to be extremely or very important. Furthermore, all factors related to provision of visual aids had median scores <6, even though consensus was obtained on all 6 factors. Combining the results on importance and obtained consensus, we can conclude that consensus (IQR ≤ 1) was obtained on the importance (median ≥ 6) of 11 factors (Table 3).

#### Similarities and Differences Between Experts and Potential Future Users

The results of the second round revealed that experts agreed on the importance of 24 factors, whereas the majority of potential future users agreed on the importance of 11 factors (median ≥ 6; IQR ≤ 1). The statistical tests showed a nonsignificant (*z* = –.262, *P* = .29) difference between the overall level of consensus obtained in the two groups.

Experts and potential future users both agreed on the importance of 7 factors: (1) general information, with 1 factor (the availability of information on how to attain a healthy lifestyle), (2) ease of use, with 3 factors (clear structure, easy log-in procedure, simple site design), and (3) questionnaire completion, with 3 factors (provision of a progress bar, a clearly structured questionnaire, and an option for partial completion).

 Contrary to these similarities in perceived importance, experts and potential future users’ opinions differed on 22 factors, significantly so on 19 factors (Table 4). The multivariate analysis of variance found a significant effect of group on the importance of factors related to optimal development (*F*_1,268_ = 95.95, *P* < .001, *R* ^2^ = .90. Univariate *F* tests revealed that experts rated a lively appearance, the use of visual aids, the use of color, user friendliness (optimal layout), information in the form of visual aids (health risk information), opportunities for customizing the site, availability of a helpdesk, use of comprehensive language, simple site design, clear navigation structure (optimal ease of use), provision of relevance and personal benefits of completing the questionnaire (questionnaire completion), provision of a self-monitoring tool, option for printing and downloading information, stimulation of revisits, and iterative feedback (additional services) as significantly more important factors than did potential future users.

Conversely, potential future users rated the availability of bread crumb navigation (optimal ease of use), the provision of a guarantee that questionnaire completion will not result in receiving spam, and usage of original questions in questionnaires (questionnaire completion) as more important than did experts.

**Table 4:** Univariate F tests for differences in rating the importance of a factor between experts and users

Factor		Mean experts (n = 60)	Mean users (n = 210)	*F*	*η*^2^
**Optimal layout**					
	Lively appearance	5.48 (0.83)	5.01 (0.98)	11.37***	.04
	Professional appearance	5.87 (0.97)	5.62 (0.81)	3.84	.01
	Use of visual materials, such as pictures, videos, and graphics	5.98 (0.89)	5.21 (0.98)	30.23***	.10
	Use of color	5.55 (0.89)	5.00 (1.05)	13.85***	.05
	User friendly	6.68 (0.57)	6.09 (0.89)	24.08***	.08
**General information**					
	Information on pros and cons of a healthy lifestyle	5.53 (0.91)	5.36 (0.97)	1.58	.01
	Information on health risk behaviors	5.60 (0.81)	5.43 (0.96)	1.59	.01
**Health risk information**					
	Information in the form of visual aids, such as graphs	5.71 (1.09)	4.88 (1.06)	28.53***	.10
**Optimal ease of use**					
	Availability of a function to customize the site for personal needs	5.78 (0.98)	3.92 (1.36)	97.63***	.27
	Availability of a helpdesk	5.68 (0.89)	5.04 (1.06)	18.09***	.06
	Use of comprehensive language	6.50 (0.70)	6.01 (0.91)	14.61***	.05
	Availability of bread crumb navigation	5.05 (1.19)	5.63 (0.97)	15.22***	.05
	Simple site design	6.02 (0.89)	5.65 (0.89)	7.88**	.03
	Clear navigation structure	6.57 (0.75)	2.18 (0.95)	1084.30**	.80
**Questionnaire completion**					
	Information on relevance of questionnaire completion	5.77 (0.89)	5.51 (0.89)	3.92*	.01
	Information on personal benefits of completion	5.78 (0.92)	5.34 (0.98)	9.95**	.04
	Guarantee that completion will not result in receiving spam	5.87 (1.16)	6.29 (0.95)	8.44**	.03
	Use of original questions	4.53 (1.19)	5.59 (1.01)	46.50***	.15
**Additional services**					
	Tool to self-monitor personal health behavior change	5.88 (0.85)	4.84 (1.21)	39.41***	.13
	Opportunity to print or download relevant information	6.18 (0.83)	5.41 (1.15)	23.41***	.08
	Opportunity to regularly revisit the site	6.02 (0.89)	5.15 (1.13)	29.95***	.10
	Iterative feedback during revisits	5.92 (0.79)	5.04 (1.07)	34.45***	.11

*P < .05, **P < .01, ***P < .001.

## Discussion

The public health impact of Internet health communication programs is suboptimal in the target population [17,18,46]. Since program development is one of the main processes to influence reach of a new product [25], this study attempted to give both experts and users a say in the development process. By means of a Delphi study, we identified an extensive list of factors potentially related to optimal development of health-related websites, as well as the extent to which experts and users agreed on the importance of these factors. In addition, we deduced similarities and differences in perceived importance between experts and potential users.

### Main Findings

This study identified an extensive list of factors that might contribute to the development of health-related websites. The importance of a selected set of these factors was stressed by both experts and potential users. Developers should therefore attempt to take these factors into account when developing a health-related website. In particular, the provision of information on attaining a healthy lifestyle was emphasized. Furthermore, a clearly structured website with a simple design and the presence of an easy log-in procedure were brought up as important factors, which corresponds to results from earlier studies [31,39]. Also, with respect to optimizing questionnaire completion, several important factors were identified. To increase questionnaire completion, websites should offer a progress bar and an option for partial completion. These results are also in line with previous findings [47]. Since questionnaire length seems to be inversely related to actual participation as well as completion [48], providing an option for partial completion might be a solution to prevent attrition when using extensive questionnaires. Besides factors agreed upon by both groups, results also indicated that the majority of experts and users significantly differed on the importance of several factors. Due to their specific knowledge on theories in their area of expertise, experts should be involved in the development process to ensure scientific input. However, opinions of potential users must not be neglected because, by engaging users in this process, their involvement will increase. This involvement will subsequently increase the chances of obtaining higher levels of appreciation, prolonged and sustained use, and lower levels of attrition [37].

The recently introduced Internet intervention model [35] offers an opportunity to classify important factors identified in this study into several main areas that determine how the website is developed and functions. The first area pertains to the *appearance* of the website, which is one of the first website characteristics visitors are confronted with. Since previous research indicated that more than half of all website visitors are inclined to leave the website within the first 30 seconds [49], the exterior of the website should be appealing in order to attract sufficient visitor attention and prevent early disengagement. In this study, experts highlighted important factors, such as a lively appearance, the use of color, and the use of visual aids, as contributing to the appearance of health-related websites. Furthermore, potential users stressed the importance of developing websites with a professional appearance [39]. The model also emphasizes *behavioral prescription*, which refers to the instructions users receive on how to change their lifestyle. In the current study, both experts and potential users emphasized the importance of offering information on how to achieve a healthy lifestyle on health-related websites. Another important area of the website is the size of the *burden* that actual use of the website entails. Results from this study indicated that both experts and users value the presence of an easy log-in procedure. Furthermore, experts stressed the importance of developing a user-friendly website, to decrease the effort visitors must invest in navigating the website. This also entails a simple site design and a clear navigation structure. Furthermore, potential users indicated they appreciated the presence of bread crumb navigation and a search engine, to ensure visible navigation on the website [50]. *C* *ontent* that is provided on the website should be accurate, complete, and readable [2,51]. In line with these findings, experts agreed on the importance of using comprehensive language on health-related websites. Since health-related websites mainly aim at helping or assisting people to adopt a healthy lifestyle, experts stressed that detailed information on the pros and cons of a healthy lifestyle and on health behaviors in general should be included in the website’s content [31,39]. Additionally, potential users indicated the importance of an opportunity to download or print relevant information that is provided on the website. *Participation* is considered as another important component of the website and focuses on its ability to engage and involve visitors. Providing reinforcement is considered an important strategy to engage visitors and can be a reward for progressing through the website content [35]. The provision of personalized feedback regarding the status of lifestyle behaviors and the opportunity to self-monitor behavior change can be regarded as a form of reinforcement. In line with this, experts stressed the importance of providing a self-monitoring tool to allow visitors to assess their current health behavior status and to track their progress. Finally, *assessment* refers to the website’s ability to adjust the website to specific user needs and wishes. In this study, experts indicated that an opportunity to adjust the website to personal preferences was highly appreciated. Therefore, visitors should have an opportunity to adjust not only the appearance of the website but also its content, corresponding to their needs and wishes. Tailoring is considered to be an appropriate strategy to adjust health information to personal characteristics and preferences [52]. In addition, previous studies indicated that providing personalized, iterative feedback regarding one’s health behavior might in itself stimulate revisits to the program [31]. In line with these previous findings, the majority of experts agreed on the importance of stimulating revisits and using iterative feedback. To adjust information to personal characteristics and specific user needs, questionnaires are often requisite to obtain detailed information on these topics. As stressed in the introduction, these questionnaires often tend to be extensive and therefore sensitive to early dropout. It is therefore imperative to stimulate questionnaire completion in order to optimize adjustment to visitor’s needs and wishes. Experts indicated that information on relevance and personal benefit [47] of completing a questionnaire should be provided. Potential users indicated that developers should develop original questions and guarantee that questionnaire completion will not result in receiving spam.

The Internet Intervention model emphasizes two additional areas that were also identified in this Delphi study: mode of *delivery* and the *message*. Mode of delivery refers to ways in which the intervention content can be delivered to visitors. Specific strategies that can be used pertain to the use of animations, audio, video, or testimonials. The message area, on the other hand, focuses on the source and style of the message and addresses issues such as the trustworthiness and expertise of the website developers. Even though several factors pertaining to these areas were identified in the Delphi study, the majority of both experts and potential users did not agree on the importance of these factors.

### Limitations

Several limitations to this study should be considered. First, the development of health-related websites is a very broad topic entailing diverse elements. To obtain detailed information on factors related to each separate element of the development process (eg, deciding on layout, content, and additional services), the questionnaire was divided into 7 categories. This rather broad setup, which is often inherent to the Delphi process, may have limited the specification of in-depth information. As the Delphi method does not allow for further specification of such factors in later rounds, still more in-depth examination of such factors is required. Second, response rates for the expert study ranged between 33% and 53%. Although this range is somewhat low, previous Delphi studies have reported similar response rates [31,43,53]. Suboptimal response rates in our study might have been a consequence of experts being invited to participate in at least two rounds of the Delphi study. Especially, response rates among experts with a practice-based background, coming from the field of technical Web design, were low during the first round. To account for these suboptimal response rates and to balance the input from the various expert fields, we put additional effort into recruiting these experts for participation in the second round. Finally, to ensure dispersion and coverage of answers, as well as optimal participation rates, it is recommendable for future studies to limit the number of questions used in the Delphi study. Incorporating a small number of questions in the first-round questionnaire may allow experts to give more in-depth input and may increase participation in subsequent rounds, thereby diminishing attrition rates.

### Implications

The currents study is a first step in increasing exposure rates of online health promotion programs. The results of this study need further experimental testing to identify which (combination of) factors ultimately results in the best result. Although the vast number of factors that play a role may hinder the feasibility of a full factorial experimental design, in-depth experimental studies on the importance of some categories of factors are recommended.

## Multimedia Appendix 1

Results of the Delphi study per factor for experts and potential future users (second and third round).

## Abbreviations

IQR: interquartile range
