# Supplementary material for: Identifying Factors for Optimal Development of Health-Related Websites: A Delphi Study Among Experts and Potential Future Users
Source: J Med Internet Res. 2012 Feb 14;14(1):e18. doi: 10.2196/jmir.1863 (PMC3374541; doi:10.2196/jmir.1863)
Supplement: Supplementary file 1 [file jmir_v14i1e18_app1.pdf]

Multimedia Appendix 1: Results of the Delphi study per factor for experts and potential future users (second and third round).

Factor[a]	Second round				Third round	
	Experts N=60		Users N=120		Experts N=32	
	Mdn	IQD	Mdn	IQD	Mdn	IQD
According to your opinion, which factors determine optimal lay-out of a health related internet site?						
1. lively appearance	6	**1**	5	**1**	-	-
2. professional appearance [c d]	6	2	6	**1**	6	**1**
3. limited amount of distractions, such as advertisements or banners	5	**1**	6	2	-	-
4. possibility to customize lay-out to personal preferences	5	2	3	**1**	4	2
5. limited amount of text	5	**1**	4	**1**	-	-
6. use of visual materials, such as pictures, videos and graphics [b]	6	**1**	5	**1**	-	-
7. use of colors [b]	6	**1**	5	2	-	-
8. emphasis on healthy lifestyle	5	2	5	2	5	2
9. limited amount of themes	5	**1**	5	**1**	-	-
10. user friendly [b]	7	**1**	6	2	-	-
According to your opinion, what kind of general information should definitely be provided on health						
11. information on pros and cons of a healthy lifestyle [b]	6	**1**	5	**1**	-	-
12. information on how to obtain an healthy lifestyle [b d]	6	**1**	6	**1**	-	-
13. information on the trustworthiness of developers	5	2	5	**1**	5	2
14. personal –tailored- information on health [c]	6	2	5	**1**	6	**1**
15. information on diseases	5	**1**	5	**1**	-	-
16. information on health risk behaviors [b]	6	**1**	5	**1**	-	-
17. results of scientific studies in the field of health and health behavior and healthy lifestyle	5	**1**	5	**1**	-	-
18. the latest news on health related topics	5	2	5	**1**	5	2
19. links to other relevant websites	5	**1**	5	**1**	-	-
20. testimonials / exemplary cases	5	**1**	5	2	-	-
21. patient information related to health and health behaviors	5	**1**	5	2	-	-
According to your opinion, what kind of health risk information should definitely be provided on health related internet sites?						
22. information on pros and cons of health risk behaviors	5	**1**	5	**1**	-	-

23. information on the relation between health (risk) behaviors and health risks	5	**1**	5	**1**	-	-
24. tailored information on personal health risks	5	**1**	5	**1**	-	-
25. information on methods to compute personal health risks	5	**1**	5	**1**	-	-
26. information on skills that help to decrease health risks	6	2	5	**1**	6	2
27. testimonials / exemplary cases	5	**1**	5	**1**	-	-
28. personal advices on how to decrease health risks	6	2	5	**1**	6	2
29. opportunity to monitor changes in personal health risks	5	**1**	5	**1**	-	-
30. positive reinforcement in case of low or decreased health risks	5	**1**	5	**1**	-	-
31. information on prevalence rates of common diseases	5	2	5	**1**	5	2
32. information compromising visual aids, e.g. graphs [b]	6	**1**	5	**1**	-	-
33. information on professional organizations in the field of health risks	5	2	5	**1**	5	2
34. information on specific health (risk) behaviors related to health risks	5	**1**	5	**1**	-	-
35. information on the trustworthiness of developers	5	**0**	5	**1**	-	-
36. information on health risk status of relevant others to allow for comparison	5	**1**	5	2	-	-
37. personal feedback on potential changes in health risks	5	**1**	5	2	-	-
Which factors determine optimal ease of use of health related internet sites, according to your opinion?						
38. clear structure [b] ‡	7	**1**	6	**1**	-	-
39. availability of a forum	4	2	5	**1**	4	2
40. availability of a chat function	4	1	4	2	5	2
41. availability of interactive components, e.g. games and test	5	2	4	2	5	2
42. limited amount of links to other (relevant) websites	4	**1**	4	**1**	-	-
43. availability of a library containing relevant information on health and health behaviors	5	2	5	**1**	5	3
44. availability of a function to customize the site for personal needs [b]	6	**1**	4	2	-	-
45. availability of contact information from developers [c]	6	2	5	**1**	6	**1**
46. availability of an easy log-in procedure [b d]	7	**1**	6	**1**	-	-
47. availability of a helpdesk [b]	6	**1**	5	2	-	-

48. little amount of scrolling required to navigate the site	5	**1**	5	2	-	-
49. use of comprehensive language [b]	7	**1**	6	2	-	-
50. availability of a search engine	6	2	6	2	6	2
51. availability of bread crumb navigation [d]	5	2	6	**1**	5	2
52. limited amount if distractions, such as advertisements and pop-ups	6	2	6	2	6	2
53. availability of a function to tailor available information to personal needs	5	2	4	**1**	5	2
54. simple site design [b] [d]	6	**1**	6	**1**	-	-
55. clear navigation structure [b]	7	**1**	2	2	-	-
According to your opinion, which factors determine whether visitors complete questionnaires provided on health related internet sites?						
56. provide information on relevance of questionnaire completion [b]	6	**1**	5	**1**	-	-
57. provide incentives for completion	5	2	5	**1**	5	2
58. provide information on personal benefits of completion [b]	6	**1**	5	**1**	-	-
59. provide a progress bar [b] [d]	6	**1**	6	**1**	-	-
60. provide clearly structured questionnaire by using clear heads and subheads [b] [d]	6	**1**	6	**1**	-	-
61. provide guarantee that completion will not result in the receipt of spam [d]	6	2	7	**1**	5	**1**
62. provide personalized feedback on questionnaire results after completion	5	2	5	**1**	5	2
63. divide extensive questionnaires into several shorter parts	5	**1**	5	2	-	-
64. provide opportunity to stop completion and proceed at a later point in time [b] [d]	6	**1**	6	**1**	-	-
65. provide guidance during questionnaire completion	5	2	5	2	5	2
66. use of original questions [d]	4	**1**	6	**1**	-	-
What kind of visual aids should be provided on health related internet sites, according to your opinion?						
67. provide cartoon	4	**1**	4	**1**	-	-
68. provide pictures	5	**1**	5	**1**	-	-
69. provide videos to replace written text	5	2	5	**1**	5	**1**
70. provide graphical representations of relevant information	5	**1**	5	**1**	-	**-**
71. refrain from using visual aids	2	3	4	**1**	2	2
72. provide opportunity for visitors to determine the use of visual aid	5	2	5	**1**	4	2

What additional services should be provided on health related internet sites, according to your opinion?						
73. provide a self-monitoring tool to monitor personal health behavior change [b]	6	**1**	5	2	-	-
74. provide an ask-the-experts section	5	**1**	5	**1**	-	-
75. provide opportunity to communicate in public with other visitors, e.g. discussion board, forum	5	**1**	4	2	-	-
76. provide opportunity to communicate in private with other visitors, e.g. chat	4	**1**	4	2	-	-
77. provide the opportunity to start a personal blog	4	**1**	4	2	-	-
78. provide contact information from developers	5	**1**	4	**1**	-	-
79. provide information on the working of the site	5	2	5	**1**	4	3
80. provide a privacy statement [c]	6	2	5	2	6	**1**
81. provide the opportunity to print or download relevant information [b]	6	**1**	5	**1**	-	-
82. provide links to other relevant websites	5	**1**	5	**1**	-	-
83. provide a search engine	5	**1**	6	**1**	-	-
84. provide the opportunity for regular revisits to the site [d]	6	**1**	5	**1**	-	-
85. provide iterative feedback during revisits to assess users against their own previous performances [b]	6	**1**	5	2	-	-

Mdn, median score

[a] Only experts were included in the third round. Statements on which consensus was obtained in the second round were excluded from the third round and results are therefore missing and indicated with a line. The bold values represent the factors on which consensus was obtained (IQD $\leq$ 1). [b] indicates items on which consensus was obtained in the second round by experts and which had a median score $\geq$ 6. [c] indicates items on which consensus was obtained in the third round by experts and which had a median score $\geq$ 6. [d] indicates items on which consensus was obtained in the second round by potential future users and which had a median score $\geq$ 6.
